# Decision Making assessed by the Iowa Gambling Task and Major Depressive Disorder A systematic review

**DOI:** 10.1590/1980-57642018dn12-030005

**Published:** 2018

**Authors:** Alaise Silva Santos de Siqueira, Mariana Kneese Flaks, Marina Maria Biella, Sivan Mauer, Marcus Kiiti Borges, Ivan Aprahamian

**Affiliations:** 1 Division of Geriatrics, Department of Internal Medicine, University of São Paulo Medical School, SP, Brazil.; 2 Institute and Department of Psychiatry, Faculty of Medicine, University of São Paulo, SP, São Paulo, Brazil.; 3 Department of Internal Medicine, Faculty of Medicine of Jundiaí, Jundiaí, SP, Brazil.

**Keywords:** major depressive disorder, decision-making, neuropsychology, Iowa Gambling Task, systematic review, transtorno depressivo maior, tomada de decisão, neuropsicologia, Iowa Gambling Task, revisão sistemática

## Abstract

Major Depressive Disorder (MDD) can occur in parallel with cognitive impairment. The search for a neuropsychological profile of depression has been pursued in the last two decades. However, scant research has been done on executive functions and decision-making ability (DM).

**Objective:**

To perform a systematic review of the evidence of DM performance evaluated using the Iowa Gambling Task (IGT) in adults with MDD.

**Methods:**

A systematic search according to the PRISMA statement was performed on MEDLINE for studies in English using the following keywords: ‘depression’, ‘depressive’, ‘depressive symptoms’ AND ‘decision making’ OR ‘game task’.

**Results:**

Five articles that met the inclusion and exclusion criteria were identified. Three reported significant differences between depressed and non-depressed individuals. The results indicated that young adults with MDD exhibited lower performance on all or almost all stages of the IGT. One study that evaluated DM in older adults with MDD showed that depressed non-apathetic participants failed to adopt any advantageous strategy and continued to make risky decisions during the task.

**Conclusion:**

Results suggest that performance on the DM task by young and old adults with MDD differed in comparison to non-depressed participants. Given the small number of articles, further studies should be performed.

## Resumo

O Transtorno Depressivo Maior (TDM) pode ocorrer em paralelo com o comprometimento cognitivo. A busca por um perfil neuropsicológico da depressão tem sido perseguida nas últimas duas décadas. No entanto, poucas pesquisas foram feitas sobre funções executivas e capacidade de decisão (DM).

**Objetivo:**

Realizar uma revisão sistemática das evidências do desempenho do DM avaliado pela Iowa Gambling Task (IGT) em adultos com TDM.

**Métodos:**

Uma pesquisa sistemática de acordo com a declaração PRISMA foi realizada na MEDLINE para estudos em inglês usando as seguintes palavras-chave: “depressão”, “depressivo”, “sintomas depressivos” e “tomada de decisão” OU “tarefa de jogo”.

**Resultados:**

Foram identificados 5 artigos que atenderam aos critérios de inclusão e exclusão. Três relataram diferenças significativas entre indivíduos deprimidos e não deprimidos. Os resultados indicaram que os jovens adultos com TDM exibiram menor desempenho em todos ou quase todos os estágios da IGT. Um estudo que avaliou o DM em idosos com TDM mostrou que os participantes deprimidos não apáticos não adotaram nenhuma estratégia vantajosa e continuaram tomando decisões arriscadas durante a tarefa.

**Conclusão:**

Os resultados sugerem que o desempenho na tarefa de DM por jovens e adultos idosos com TDM diferiu em comparação com os participantes não deprimidos. Dado o pequeno número de artigos, mais estudos devem ser realizados.

Depression is a known risk factor for cognitive decline.^1^^,^ 2 The opposite is also true, since cognitive impairment is a risk factor for major depressive disorder (MDD).^3^ No single cognitive function has been found to characterize all depressed patients; and also, not all patients have impairment to the same cognitive domain or to the same degree.^1^ Nonetheless, it has been found in many studies with depressed patients that memory is overvalued and better investigated than other cognitive abilities such as attention, executive functions, and speed of information processing.^4^ Indecision is also included in the DSM-5 criteria for MDD^5^ defined as difficulty to think and/or concentrate as a cognitive symptom of depressive disorder. Additionally, the ICD-10,^6^ the Beck Depression Inventory (BDI),^7^ and other instruments that evaluate the diagnosis of depression also consider the presence of indecision.^8^ Depressed patients generally report more decision-making problems compared to healthy subjects. It is therefore necessary to investigate this depressive symptom, that has not received much attention and clinical investigation.^9^

Decision Making (DM) can be defined as the process of choosing between two or more competing alternatives, which require cost analysis and critical thinking about the benefit of each option and estimation of its consequences in the short, medium, and long terms.^10^ DM is part of the executive function and is a cognitive ability related to the capability to evaluate the environmental information associated with a given choice, which will ensure that actions are taken after analyzing the positive and negative aspects of each option.^11^ DM can be studied experimentally using tasks that expose individuals to fictitious games. The most used instrument worldwide in the evaluation of ambiguity scenarios is the Iowa Gambling Task (IGT).^12^^,^ 13 The IGT is a computerized task (deck of cards) in which the participant must choose between four different decks. Although it is not made explicit to the participants, two of the four decks are advantageous and two are disadvantageous. Decks are considered advantageous when the immediate gain is low, but in the long run the punishment is also low. A negative profile is assumed when the immediate reward is high, but the punishment (loss of money) is also high in the long run. In other words, the rules about gains and losses are not explained. Thus, in order to solve the task successfully, participants must discover implicit rules in the feedback they receive after each choice (for more details see Bechara).^13^^,^ 14

As illustrated above, it is very important to understand the relationship of the DM process in the context of MDD. However, there is a dearth of studies addressing this topic and those that exist do not reach a consensus in the current literature. Therefore, the aim of this systematic review was to provide an overview of the existing research in the area of DM using IGT for the evaluation of both young and older adults with MDD.

## METHODS

**Figure 1:**
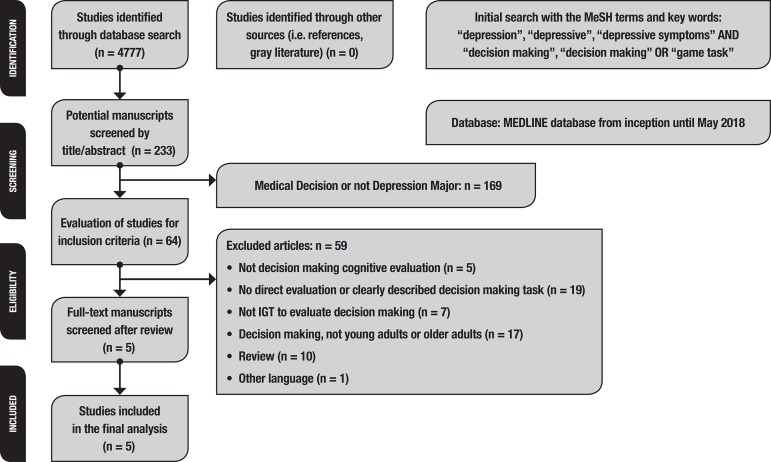
Flow diagram of the systematic search.

A systematic review of a DM task and MDD was conducted from inception to May, 10^st^ 2018. A computerized search for studies published in English was carried out on PubMed (MEDLINE) using the following MeSH terms and keywords: ‘depression’, ‘depressive’, ‘depressive symptoms’ AND ‘decision making’ OR ‘gambling task’. This initial search yielded 4777 references. We included 233 studies after restriction of the MeSH terms for title and abstract content. A manual search further identified studies according to the following inclusion criteria: original studies, studies available in English, not letters to the editor, and editorials or reviews; resulting in the retrieval of 64 articles. The exclusion criteria included current or previous bipolar disorder, schizophrenia, psychosis, substance abuse, dementia, and neurological disease, including head trauma. Suicide attempts within last 2 years were also part of the exclusion criteria. A further analysis was carried out in which only studies whose main objective was to evaluate the performance of DM using the IGT were included. Seven studies were excluded giving a final total of 5 studies of DM and MDD selected according to the inclusion criteria (Table 1). Study selection was based on the agreement between two authors using the Preferred Reporting Items for Systematic Reviews and Meta-Analyses (PRISMA; www.prisma-statement.org) statement checklist and flow diagram as a reference for quality analysis (A.S. and M.M.B). Study selection was reviewed and analyzed by the other authors (I.A. and S.M). The flowchart of study selection is depicted in Figure 1.

**Table 1:** Summary of systematic review on Major Depressive Disorder and decision-making ability.

Author, year, location	Sample	Aim of the studies	Diagnostic Criteria	Instruments for evaluation of DM	Results	Comments
Must et al., 2006, Hungary	50 participants 20 controls, mean age 42.5 30 depressive individuals mean age 43.8	Evaluation of DM(1) and EF(2) in MDD(3) on two versions.	Mini-International Neuropsychiatric Interview, HAM-D(4)	IGT(5) Bechara et al.,1994	Patients with MDD were impaired on the WCST(6) and on the ABCD version of the IGT but showed normal performances on the EFGH task.	Limitations: The sample size was small and only few neuropsychological tests were used. Unmedicated patients were not assessed. Individual personality style, response strategies, and behavioral impulsivity were not investigated.
Smoski et al., 2008, USA	85 participants 44 controls, mean age 36.7 41 depressive individuals mean age 37.9	Examine whether depression is associated with greater responsiveness to negative feedback in relation to reward.	HAM-D 17-item version	IGT Bechara et al.,1994	Depressive participants chose fewer risky cards across the entire task compared to control participants and showed a trend toward winning more money overall.	The design of the present study makes it difficult to discern whether IGT performance among depressive individuals was superior due to a heightened response to punishment, a decreased response to reward, or both.
Cella et al., 2010, United Kingdom	39 participants 20 controls, mean age 35.1 19 depressive individuals mean age 35.8	Explore flexible DM performance in patients with MDD with the contingency shifting variant IGT.	BDI-II(7)	Contingency shifting variant IGT Dymond et al., 2010	Show impaired performance by MDD patients on all phases of the IGT, relative to control participants, and emphasizes the role that altered sensitivities to reward and punishment may play in the impaired DM often found in depression.	Limitations: sample size was relatively small. Two of the nineteen MDD patients were receiving adjuvant mood stabilizer medication, which may have impaired performance. The findings can only provide limited insight into the effect of antidepressant medication on flexible DM in depression due to the absence of a group of unmedicated MDD patients.
McGovern et al., 2014, NY	96 participants 36 controls, 60 depressive individuals (age >60)	To identify abnormalities in reward- related DM in late-life depression.	DSM-IV-TR criteria, Research Diagnostic Criteria for unipolar major depression, HDRS 24-item	IGT Bechara et al.,1994	Apathetic depressed older adults more effectively evaluated costs and benefits and shifted their selections to the conservative decks. In contrast, non-apathetic, depressed older adults did not adopt an advantageous strategy and continued to make risky decisions on the task.	Limitations: All groups demonstrated deteriorating performance on later time blocks of the IGT. There is no unimpaired reference group in this study. Furthermore, since the IGT is one of several well-studied DM tasks, it is important to determine whether other complex DM tasks reveal similar patterns.
Moniz et al., 2016, Portugal	60 participants 30 controls mean age 41.43 30 depressive individuals mean age 42.20	To compare the performance of a group of 30 non-psychotic unipolar depressed against 30 healthy controls on a version of the IGT	MINI(8); BSI(9)	IGT Mueller & Piper, 2014	Significant differences were found between depressed patients and healthy controls in traditional Net Score measures as well as in various alternative metrics.	Limitations: sample size, regarding both patients group and health control.

(1) Decision-making,

(2) Executive functions,

(3) Major Depressive Disorder,

(4) Hamilton Depression Rating Scale,

(5) Iowa Gambling Task,

(6) Wisconsin Sorting Card Test,

(7) Beck Depression Inventory,

(8) Mini International Neuropsychiatric Interview,

(9) Brief Symptom Inventory.

## RESULTS

Of the 5 studies included, 4 evaluated DM in young adults with MDD and 1 in older adults. Geographic heterogeneity can be observed in these studies, since 2 studies took place in the USA,^15^^,^ 16 1 in Hungary,^17^ 1 in the United Kingdom,^18^ and 1 in Portugal.^19^ The samples of all of the selected studies can be considered relatively small, ranging from 39 to 96 participants. Patients and controls did not differ significantly in relation to gender, age or education level in each study.

The diagnosis of depression was established based on different symptomatic scales. A single study used the DSM-IV criteria.^16^ The most used scales were the Hamilton Depression Scale (HAM-D)^15^^-^ 17 and the Mini International Neuropsychiatric Interview.^17^^,^ 19 The study that evaluated DM in MDD in older adults^16^ also evaluated apathy using the Apathy Evaluation Scale (AES).^20^

There were discrepancies in the use of IGT versions. Three studies used the original form.^15^^-^ 17 One of them^17^ also used the modified version (EFGH).^21^ Unlike the original version, this version requires the choice of decks in which immediate losses are high, but the rewards are also high. In this version, it is also inferred that the subject may exhibit hypersensitivity to the reward. Another study^19^ used a computerized IGT similar to the commercial version.^22^ In the latter study, a version of the IGT with more trials was used.^18^ In the study, IGT was divided into 2 phases: 100 trials (phase 1) followed by 120 trials (phase 2). In phase 1 the IGT was applied in its original form, but in phase 2 three trials of contingency were introduced. Instead of phase 1, the beginning of other phases was not indicated, and involved a progressive modification of contingencies of reward and punishment (more details in Dymond).^23^

There were no uniform results among DM studies in patients with MDD. Must et al. (2006) found that patients with MDD took less advantageous decisions than controls using the IGT ABCD version, after 41-60, 61-80, and 81-100 trials (t<-3, p<0.001, power >0.90). There were no significant differences between groups using the EFGH version. The measures from the Wisconsin Card Sorting Test, IGT, and HAM-D were not correlated (*R*<0.2).

The study by Smoski^15^ showed that all participants learned to avoid risk decks throughout the task (F (4,332)=9.60, p<0.001, η_p_^2^=0.12) with a significant decrease in the number of risky cards selected over time. Depressed patients chose fewer risky cards throughout the task (F (1,83)=4,03, p<0.05, η_p_^2^=0.05) compared to the control group, that showed a tendency to make more money in general (t (83)=1,71, p=0.09).

Cella^18^ showed that controls had better performance on all IGT blocks compared to the MDD patients in phase 1 (all *p*<0.05). In phase 2, controls performed better on blocks 7, 9, 10, and 11 (*p*<0.05). Finally, Moniz^19^ observed a significant difference between depressed patients and controls in the total Net Score Measurement (t= -3.852, df=58, p=0.001, d= -994), and also in the alternatives metrics variables CD-AB 21-100 (t= -.2.873, df=100, p=0.005, d= -.569) and CD-AB 41-100 (*p*=0.005).

The study by McGovern^16^ involving a sample of older adults showed no significant differences in terms of performance throughout the task (F (3.57, 335.36)=0.49, *p*=0.73) in comparison of depressed patients versus healthy controls. They also did not differ significantly in deck preferences (t (94)=0.40, *p*=0.69) or sensitivity in frequency of punishment (t (94)= -0.43, *p*=0.67). However, in the comparison between apathetic depressed subjects and depressed non-apathetic subjects, the former group selected significantly (t (58)=3.0, *p*<0.05) fewer cards from the risk deck (A and B) than the latter group. Moreover, apathetic patients selected significantly (t (58)= -3.0, *p*<0.05) more cards from the conservative decks (C and D) compared to depressed non-apathetic patients. As a global measure of performance of the whole task, the apathetic group made significantly more advantageous decisions (t (58)= −3.0, *p*<0.05) and earned much more money than the depressed non-apathetic group. On the other hand depressed non-apathetic older adults did not embrace an advantageous strategy and continued to make risky decisions on the task.

## DISCUSSION

The aim of this systematic review was to investigate the DM process using the IGT in individuals with MDD. The results of the studies were not uniform. Of the 5 studies included in this review, 3 reported significant differences between depressed and non-depressed subjects.^17^^-^ 19 These results indicated that younger adults with MDD had altered sensitivity to reward or punishment, since they exhibited impaired performance in all or almost all phases of the original IGT version. Smoski^15^ reported different results from these 3 studies, as their depressed participants chose fewer risky cards throughout the task compared to the control group. This sample also demonstrated a tendency to make more money overall. However, in this study, it is hard to discern whether IGT performance among depressed participants was higher because of a heightened response to punishment, a decreased response to reward, or both.

The study that evaluated DM in older adults with MDD showed that depressed apathetic patients evaluated a cost and benefit balance more effectively, and then changed their selections to a more conservative deck when compared to the depressed non-apathetic group.^16^ On the contrary, depressed non-apathetic participants did not adopt an advantageous strategy and continued to make risky decisions. This result is consistent with the other 3 studies showing positive results for DM in younger adults.^17^^-^ 19 The reasons why apathetic participants had a favorable decision-making profile warrant further evaluation. This may have been due to lower engagement in trying to achieve more significant gains.

The studies evaluated in this review showed that there is no consensus in the literature on the existence of differences in DM performance among MDD patients. It is noteworthy, however, that this disparity may be a consequence of different study designs. This could be minimized if the studies had equivalent characteristics of methods and instruments.

Impairment of DM is also observed in other mental illnesses. Patients diagnosed with Borderline Personality Disorder (BPD) made less advantageous choices on the IGT than healthy controls. The impact of BPD is interesting, since depression and BPD probably share some of the same neurobiological substrates.^24^ DM was also evaluated in patients with Obsessive Compulsive Disorder (OCD).^25^ OCD patients are impaired in DM when assessed for ambiguity by IGT, but not when assessed for risk by the Game Dice Task (GDT). These findings confirm that DM processes are dissociated in OCD.

Regarding DM and aging, a systematic review was conducted comparing DM in younger and older adults.^26^ Nine studies were found. Only 2 showed significant differences between groups according to the evaluation of the general index of performance.

Little is known about the relationship of drug treatment in MDD with DM performance. To date, one study has sought to assess whether performance on cognitive control and reward-related DM tasks predict changes in symptoms and signs of MDD during treatment with selective serotonin reuptake inhibitors (SSRI).^27^ According to the results of the study, irregularities in control of cognitive tasks, but not in DM, influenced the trajectory of symptoms and also depression remission during the treatment with antidepressants.

The main limitation of the studies included in this review was small sample size, in both case and control groups, no use of a single instrument for the diagnosis of MDD, the different versions of IGT used, and the lack of other measures of executive functions. Additionally, it seems that cognitive impairment was associated with current depression medicated using antidepressants, but no clear mention was made about treatment duration, recurrent depression or psychological support.

In conclusion, the results of this systematic review suggest there is no consensus on the processes of MDD and DM in younger and older adults. The limited number of articles, makes further studies necessary to gain a better understanding of the DM process and its influences on MDD. In this regard, the use of other neuropsychological tests to evaluate this cognitive domain is also recommended. Moreover, it is important to have a better understanding of the strategies of the DM process, and about the anatomical areas involved in performing this task. These findings may have important clinical and public health implications.
